# Punishment Based on Public Benefit Fund Significantly Promotes Cooperation

**DOI:** 10.1371/journal.pone.0105126

**Published:** 2014-08-19

**Authors:** Xiuling Wang, Jie Wu, Gang Shu, Ya Li

**Affiliations:** 1 School of Computer and Information Science, Southwest University, Chongqing, China; 2 School of Physical Science and Technology, Southwest University, Chongqing, China; University of Maribor, Slovenia

## Abstract

In prisoner's dilemma game (shortly, PD game), punishment is most frequently used to promote cooperation. However, outcome varies when different punishment approaches are applied. Here the PD game is studied on a square lattice when different punishment patterns are adopted. As is known to all, tax system, a common tool to adjust the temperature of the economy, is widely used in human society. Inspired by this philosophy, players in this study would pay corresponding taxes in accordance with their payoff level. In this way, public benefit fund is established consequently and it would be utilized to punish defectors. There are two main methods for punishing: slight intensity of punishment (shortly, SLP) and severe intensity of punishment (shortly, SEP). When the totaling of public benefit fund keeps relatively fixed, SLP extends further, which means more defectors would be punished; by contrast, SEP has a smaller coverage. It is of interest to verify whether these two measures can promote cooperation and which one is more efficient. Simulate results reveal that both of them can promote cooperation remarkably. Specifically speaking, SLP shows constant advantage from the point of view either of fractions of cooperation or average payoff.

## Introduction

Early in the primitive society, humans learned to work in groups to capture prey. Even nowadays, there is no doubt that cooperative behavior exists widely in biological, social and economic systems [1]. Understanding the evolution of cooperation among unrelated individuals is still a major challenge to many natural and social scientists [2]. Thus far, evolutionary game [3]–[9] theory has provided a common mathematical framework to solve this problem. Especially the classical prisoner's dilemma (PD) game describes the conflict between individuals, it is commonly employed in biology and applied to many non-human species. Therefore its extensions have been researched widely [10]–[12].

Since the previous work conducted by Nowak and May [10], evolutionary games have been widely researched on lattices [13], [14] and complex networks [15]–[18]. In the previous studies, researchers found that popular mechanisms such as kin selection [2], [19], the time scale of strategy updating [20], [21] and spatial topology [22]–[24] played an important role in the emergence of cooperation. Recently, a simple rule in strategy changing based on the value of a single parameter 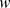, which influences the selection of players that are viewed as potential sources of the new strategy is adopted [25]. Results revealed that increasing the probability of adopting the strategy from the fittest player within reach (setting 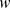 positive) promotes cooperation. Ref. [26] has researched correlation 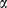 between the payoff and the increasing age of players and found that moderate values of 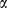 allow cooperators outcompete defectors. In [27], the time course of cooperation evolution under different evolution rules is studied. It is found that the formation of the perfect C cluster at the end of the enduring period and the expanding fashion of the perfect C cluster during the expanding are two factors to determine the final cooperation level. Ref. [28] has studied the evolution of cooperation under two different evolutionary games within a fraction 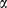 of each players' payoffs gained from direct game interactions, where 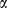 determines the degree of the relatedness among the neighboring players. It found that closer relatedness can remarkably promote cooperation in the context of both games. Moreover, Ref. [29] investigated the emergence of cooperation in square lattice when adopting Dempster-Shafter theory, which is an important tool for decision analysis and predictiton [30]–[33], to combine evaluations from the point of payoff and environment. Simulate results revealed that the comprehensive strategy updating method promotes cooperation significantly. Most recently, evolutionary games have also been studied on interdependent networks [34]–[37]. In [34], it focused on evolution of public cooperation on two interdependent networks that are connected depending on a utility function, which is used to determine to what extend payoff in one network influence the players in the other network. Results indicated that the stronger the bias in the utility function, the higher the level of public cooperation was. Ref. [35] revealed that only an intermediate density of sufficiently strong interactions between networks warrants an optimal resolution of social dilemmas. In [36], two-layer scale-free networks with all possible combinations of degree mixing are studied, one is used for the accumulation of payoffs and the other is used for strategy updating. It turned out that breaking the symmetry impedes the evolution of cooperation. Ref. [37] showed that the interdependence between networks self-organizes is helpful to yield optimal conditions for the evolution of cooperation.

Pre-existing studies reminded us the fact that defection may lead to the tragedy of the commons [38]. Aimed to overcome this unfavorable outcome, a great of measures have been identified to promote cooperation. Typical measures include reward [39], [40] and punishment [41]–[45]. Here, we focus on punishment. Nevertheless, punishment is costly. Unlike the situation in most studies in which cost of punishment is paid by cooperators [44], [46]–[50], we collect public benefit fund by charging players the corresponding taxes according to the level of players' payoff. It would be used to afford the cost of punishment. Furthermore, we also take the punishment intensity and punishment range into consideration. However, they can not be satisfied at the same time. Limited by resources, it is often the case that severe inspection system only works on a small scope, because the establishment of severe system is overwhelmed by certain resources, and vice versa. Hence there mainly exist two measures for punishment: slight intensity of punishment (SLP) and severe intensity of punishment (SEP). In this paper, we investigate whether this new plan has a positive impact on the emergence of cooperation and which pattern is more effective. Simulate results indicate that SLP shows more efficiency.

### Model

The PD game is conducted on a square lattice of size *L*×*L* with periodic boundary conditions. As a matter of rountine [51], the payoffs are listed below: *T* = *b*, where T is the temptation to defect and 1<*b*<2, *R* = 1, where R is the reward for cooperation, *P* and *S*(*P* = *S* = 0) is the punishment for mutual defection and the sucker's payoff, respectively. Although in this model of the weak PD game has *P* = *S* rather than *P*>*S*, it captures simply the essential social dilemma.

Initially, each player *x* is designated as a cooperator or defector with probability of fifty percent. At every time step, each player on the square lattice plays PD games with all four nearest neighbors and then obtains accumulated payoff *U*. Next, our studies are conducted in two different situations: The PD game without punishment and the PD game with punishment.

### The PD game without punishment

Each player *x* chooses one of its neighbors *y* randomly and revises its strategy according to the following Fermi rule [14]. Let 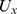 and 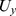 denote the accumulated payoffs of player *x* and player *y* obtained from the previous round, respectively. Player *x* adopts the neighbor's strategy with the probability 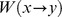,
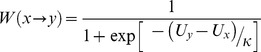
(1)where 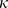 represents the amplitude of noise level, where 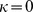 presents determinate imitation, while 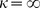 indicates stochastic imitation. In our study, we will not take the effects of 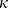 into consideration, so we set 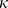 to a constant value, 0.1. Each round is started in a random initial state, with many times repeated.

### The PD game with punishment

At every time step, after obtaining accumulated payoffs by playing games with all neighbors, players' payoff is sorted in a descending order. As Table 1 shows, according to the order, players whose payoff ranks the top 25% would pay 10% of their payoff as individual tax. In the same way, players whose payoff ranks from 25% to 50% would pay 5% of payoff as individual tax. However, in accordance with reality, tax would not be charged from the players whose payoff ranks the last 50%. In this way, a certain quantity of public benefit fund can be collected and it could be used to cover the cost of punishment. At each round, we will set a punishment intensity *p*, where 0<*p*<1. That is to say, the fine that defectors need to suffer is *p*×*b*, where *b* denotes the value of temptation, which means the higher *b* is, the more penalty is. We also set a punishment range *q*, where 0<*q*<1, in other words, the number of defectors to be punished is *d*×*q*, where d denotes the total number of defectors. As mentioned above, the intensity *p* is inversely proportional to punishment range *q*. Here the simplest liner relationship is adapted to imitate: *q* = 1−*p*. The penalty with corresponding intensity would be imposed in given range at every time step. After that, payoff of players would be refreshed again. Next, Each player *x* chooses one of its neighbors *y* randomly and revises its strategy according to Fermi rule in Eq. 1. And in next step, a new round of game begins.

**Table 1:** Public benefit fund collected by the rank of payoffs.

Ranking Payoff	0–25%	25%–50%	50%–100%
Corresponding taxes	10%×*U*	5%×*U*	0

## Results and Analysis

**Figure 1:**
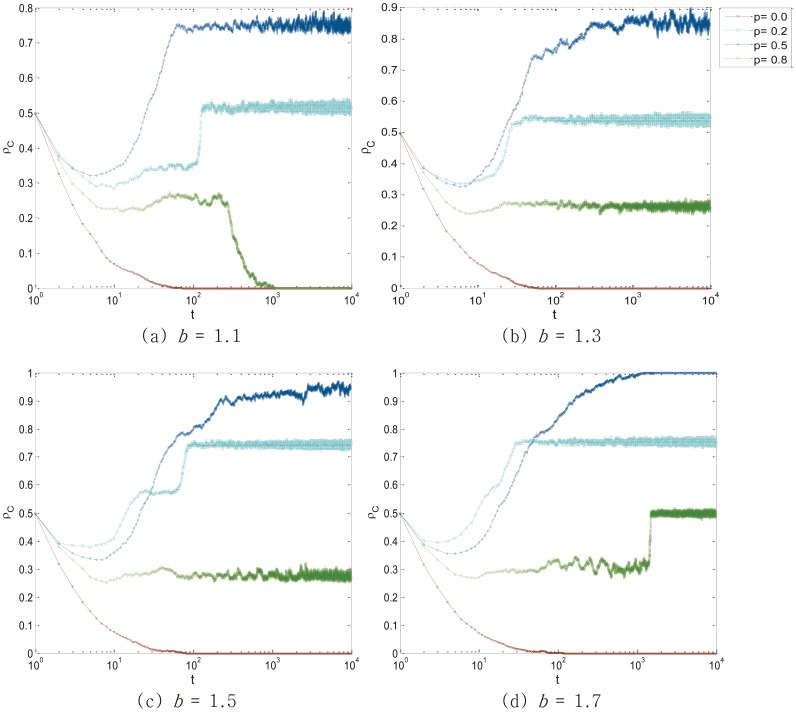
Temporal evolution of the cooperators density 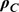 towards its stationary state for different values of *b* and different punishment intensity *p*. SUP leads to the most effective effect and SLP is better than SEP. With the increase of *b*, the punishment is more efficient. Employed parameter value was: *L* = 100.

The game is played in a square lattice of size *L* = 100. The impact of punishment on the outcome of the game can be fully understood only if the same experiments are carried out without the punishment. Therefore we first conduct the experiments in the absence of punishment to arrive at a baseline scenario, in particular to estimate the cooperators density 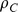 at different values of *b*. We start by investigating how the temptation to defect *b* and the punishment intensity *p* affect the evolution of cooperation. The simulate results for fraction of cooperation 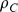 with four values of *p* are shown in Fig. 1. To investigate the effectiveness of punishment when applying different intensity *p*, we focus on *p* = 0.2, *p* = 0.5 and *p* = 0.8, which represents SLP, suitable intensity with suitable range (shortly, SUP) and SEP respectively.

Figure 1 shows that with varying values of *p*, the fraction of cooperation 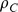 rises significantly, compared with *p* = 0 (without punishment), which indicates that the measure of punishment has a positive impact on the emergence of cooperation. However, with different values of *p*, 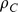 is total different. When *p* = 0.5, that is to say, not only the punishment intensity, but also the punishment range are perfect, 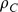 always keeps in a high level in spite of the increase of *b*. More interestingly, for *p* = 0.2 and *p* = 0.8, despite the continue increasing values of *b*, 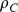 in the former conditions is always higher than those in the latter conditions. Results presented thus far indicate that the punishment offered by public benefit fund has promote cooperation. The highest 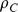 emerges when adopting SUP. However in the real world application, it is difficult to realize moderate *p*. Under such circumstance, SLP is a good choice. In other words, SLP is more suitable to promote cooperation than SEP.

**Figure 2:**
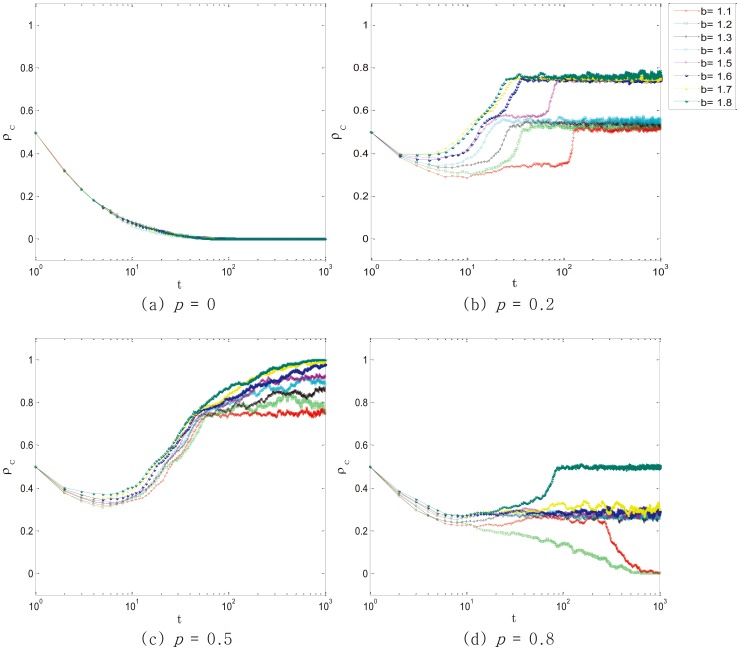
Temporal evolution of the cooperators density 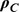 towards its stationary state for different values of *b* and different punishment intensity *p*. SUP leads to the most effective effect in all instances of *b*. According to Fig.2(b) and Fig.2(d), 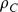 with SLP is higher than 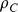 with SEP. With the increase of *b*, the punishment is more efficient. Employed parameter value was: *L* = 100.

Figure 2 provides a quantitative assessment of different values of *p* under different level of temptation to defect *b*. Obviously, 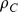 is close to 0 when *p* = 0 and 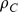 is close to 1 when *p* = 0.5. We mainly focus on *p* = 0.2 and *p* = 0.8, because these two situations are closer to reality and can be operated easier. It is not difficult to find that when *p* = 0.2 and *p* = 0.8, 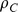 varies greatly with the values of temptation *b*. Namely, the higher *b* is, the more efficiently the punishment works. Because the penalties increase with the value of *b*, which leads to an obvious effect to promote cooperation.

**Figure 3:**
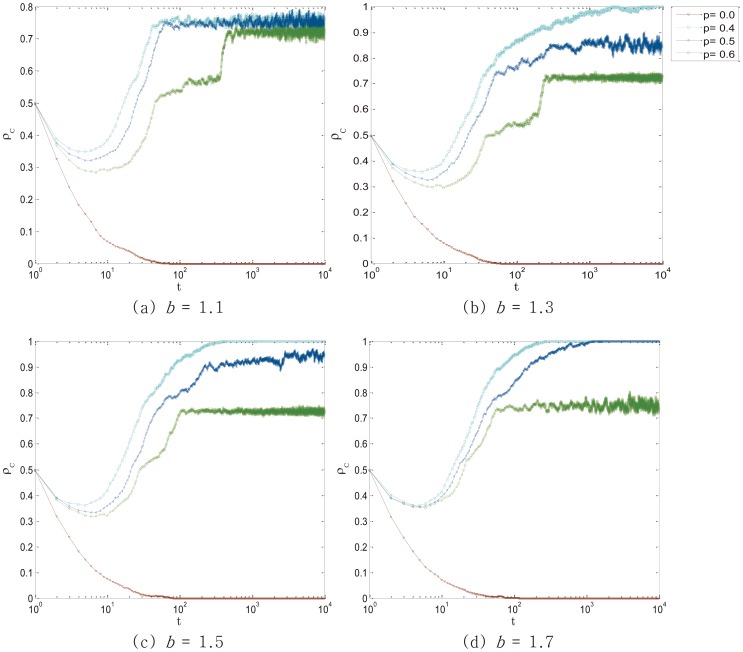
Temporal evolution of the cooperators density 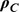 towards its stationary state for messy values of punishment intensity *p* in different values of temptation *b*. 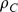
 with low *p* is always higher than 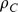 with higher *p*. Employed parameter value was: *L* = 100.

**Figure 4:**
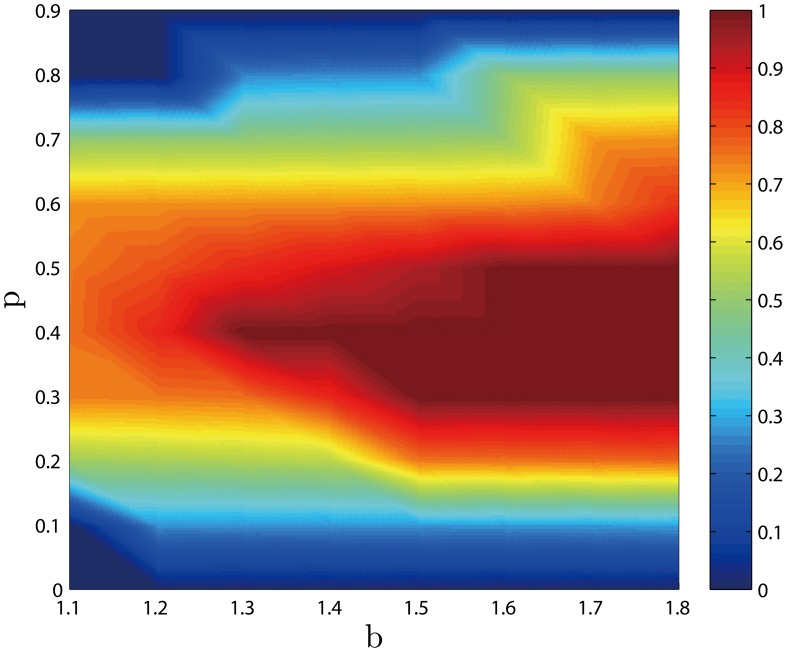
Fraction of cooperation 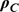 in dependence on 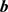 and 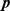. When *p* is moderate, 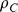 keeps in a high level. Moreover, with the increase of *b*, the average fraction of cooperation 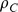 grows. When *p* is too high or too low, cooperation is gradually extinct. Employed parameter value was: *L* = 100.

Previous studies have showed that the maximal 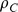 arrives at *p* = 0.5. In order to figure out whether the slight intensity is still more efficient than severe intensity under the circumstance of moderate *p*, we investigate 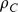 with four typical values of temptation (*b* = 1.1, *b* = 1.3, *b* = 1.5, *b* = 1.7). As Fig. 3 shows, punishment with *p* = 0.4 is most efficient, which indicates that SLP is really better than SEP. To analyze the relationship between 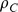, *b* and *p*, we plot the value of 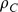 under stationary pattern in dependence on *b* and *p* in Fig. 4. It is crystal clear that the space occupied by red is dramatically increasing as a result of the rise of *b*, which indicates that 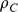 becomes higher. What is more, In circumstances of low values of *p*, the areas occupied by red is wider than that of high values of *p*. This is due to the fact that with the rise of *b*, penalties also increase, which leads to the phenomenon that defectors would pay for more fines. All this findings has proven fore-mentioned conclusions, SLP is more efficient than SEP and punishment works more effectively with the increase of *b*.

**Figure 5:**
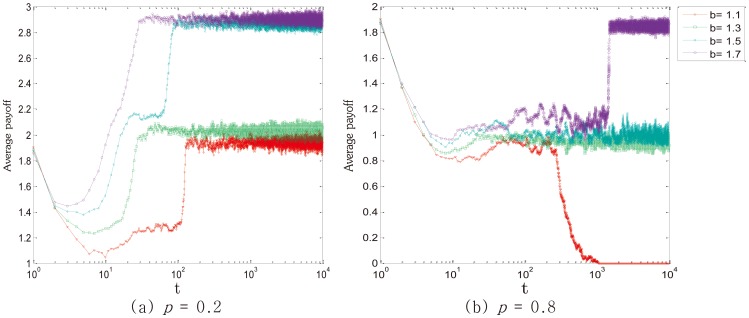
The average payoff with different values of *b* and *p*. Owing to the application of punishment, the average payoff rises significantly with the increase of *b*. Furthermore, the average payoff with SLP is always higher than that with SEP. Employed parameter value was: *L* = 100.

Moreover, we also investigate the average payoff in different situations. As is shown in Fig. 5, when applying SLP, the average payoff decreases initially and then rises slowly before reaching the steady state. It is due to that players would be charged for the public benefit fund, which leads to the drop of the average payoff. However, with the increase of 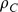, the average payoff also rises dramatically. While applying SEP, as shown in Fig. 5(b), the average payoff drops to a certain limits prior to keeping steady. It is interesting to find that the average payoff goes up when *b* sets as a high value, which proves that punishment is more effective under the circumstance of high temptation. Meanwhile, the average payoff with SLP (Fig 5(a)) is obviously higher than that with SEP. Because more defectors are punished, as a result, the cooperation is promoted. When 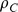 always stays in a high level, most cooperators can receive high payoffs, which improves the whole average payoff. However, in the circumstance of SEP, only few defectors are punished, players tends to defect for higher payoffs. Therefore, 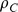 is relatively lower. On the circumstance of SEP, only few defectors get high payoffs, payoffs of most cooperators are relatively less. And it is the reason why the average payoff is lower than that with SLP. It can be conducted that SLP is better than SEP according to the average payoff.

**Figure 6:**
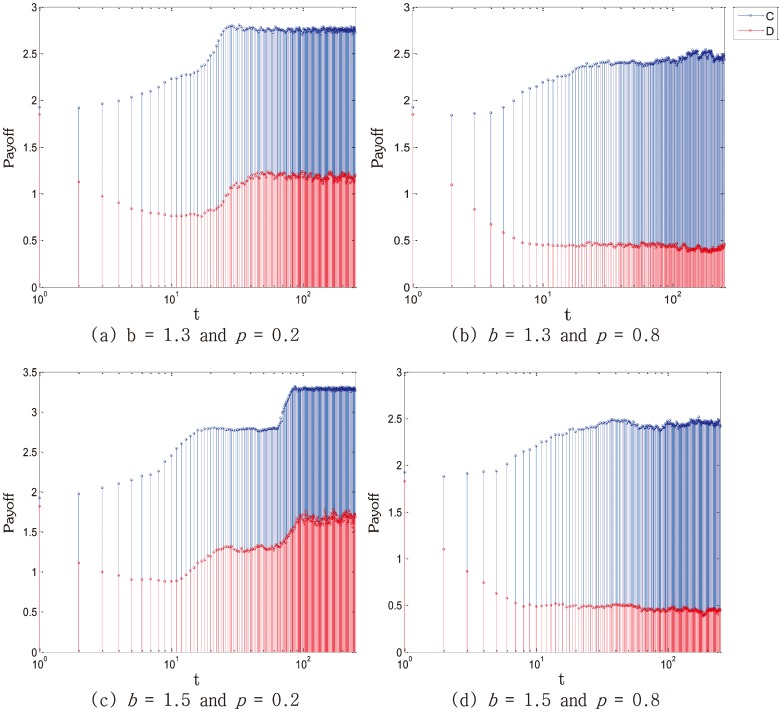
Payoff of cooperators and defectors in dependence on *b* and *p*. Cooperators are represented by blue, while defectors are indicated by red. Owing to the application of punishment and public benefit fund, average payoff of cooperators is higher than that of defectors. What's more, when applying SLP, even with the same value of *b*, the payoff is respectively higher than that applied with SEP. Meanwhile, with the increase of *b*, the payoff also rises significantly. Employed parameter value was: *L* = 100.

More detailed studies about the payoff of cooperators and defectors are carried out by Fig. 6. Overall, the payoff of cooperators is higher than that of defectors. Because cooperators form cooperative clusters and get relatively high payoff. While defectors can only rely on the profit obtained from the cooperative neighbourhood. However, their payoff will become zero if they came across with other defectors. And this is exactly the reason why the payoff of cooperators is higher than that of defectors. Furthermore, from the contrast of Fig. 6(a) and Fig. 6(c), Fig. 6(b) and Fig. 6(d), the payoff with SLP is higher than the payoff with SEP no matter are cooperators or defectors. Consequently, judging from the payoff of cooperators and defectors, SLP is still more effective than SEP.

In order to test the robustness of this observation against the change of interaction topology, the same experiment is conducted on the small world network. The mean degree is set to four, which aimed to compare with square lattice. Simulate results accord with the conclusion of this paper basically. The fraction of cooperation 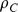 rises significantly when different values of *p* are applied, which is better than that under *p* = 0 (without punishment). The present mechanism turns out to be available to motivate cooperation in the small world network. The highest 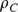 still emerges when adopting SUP. Nevertheless, with a higher value of temptation *b*, 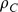 differs from that in square lattice. Regardless of varied values of *p*, cooperation is almost extinct in such situation, which means players prefer to defect for obtaining higher payoff even with the risk of being punished in the small world network.

## Conclusions

The evolutionary emergence of cooperators in social dilemmas has long been an important topic and punishment is a commonly used tool to promote cooperation. In order to provide a detailed analysis of this phenomenon, the classical prisoner's dilemma game, as a basic model, is commonly researched. In this paper, based on the imitation of taxation system, public benefit fund is collected to afford the cost of punishment. Moreover, we also take the punishment intensity and the punishment range into consideration. After a careful observation, we find that punishment intensity is in inversely proportional to range most of the time, which means there are mainly two measures for punishment: slight intensity of punishment (SLP) and severe intensity of punishment (SEP). As expected, the results have shown that this mechanism is effective, no matter from the perspective of cooperation level or perspective of average payoff. And further study also reveals the fact that if we cannot determine the most suitable intensity, slight intensity of punishment rather than severe intensity of punishment should be adopted. We also conduct the same experiment in the small world network and results follow the similar trend. It has shown that the present mechanism is robust against the change of interaction topology. A specific point that merits further research is the imitation of relation between punishment intensity *p* and punishment range *q*. In this paper, the simplest linear formula *q* = 1−*p* is adopted to simulate the inverse relation. Whether there exist a better model to describe the restrictive correlation between the two parameters needs further research. We hope that our findings may provide a reference to establish punishment system in real world.
